# SMRT–AgRenSeq-d in potato (*Solanum tuberosum*) as a method to identify candidates for the nematode resistance Gpa5

**DOI:** 10.1093/hr/uhad211

**Published:** 2023-10-17

**Authors:** Yuhan Wang, Lynn H Brown, Thomas M Adams, Yuk Woon Cheung, Jie Li, Vanessa Young, Drummond T Todd, Miles R Armstrong, Konrad Neugebauer, Amanpreet Kaur, Brian Harrower, Stan Oome, Xiaodan Wang, Micha Bayer, Ingo Hein

**Affiliations:** Division of Plant Sciences at the Hutton, The University of Dundee, Errol Road, Invergowrie, Dundee, DD2 5DA, UK; Division of Plant Sciences at the Hutton, The University of Dundee, Errol Road, Invergowrie, Dundee, DD2 5DA, UK; The James Hutton Institute, Errol Road, Invergowrie, Dundee, DD2 5DA, UK; Division of Plant Sciences at the Hutton, The University of Dundee, Errol Road, Invergowrie, Dundee, DD2 5DA, UK; College of Plant Protection, China Agricultural University, Haidian District, Beijing, 100083, China; James Hutton Limited, The James Hutton Institute, Errol Road, Invergowrie, Dundee, DD2 5DA, UK; James Hutton Limited, The James Hutton Institute, Errol Road, Invergowrie, Dundee, DD2 5DA, UK; Division of Plant Sciences at the Hutton, The University of Dundee, Errol Road, Invergowrie, Dundee, DD2 5DA, UK; Biomathematics and Statistics Scotland, Errol Road, Invergowrie, Dundee, DD2 5DA, UK; Division of Plant Sciences at the Hutton, The University of Dundee, Errol Road, Invergowrie, Dundee, DD2 5DA, UK; Crop Research Centre, Teagasc, Oak Park, Carlow R93 XE12, Ireland; The James Hutton Institute, Errol Road, Invergowrie, Dundee, DD2 5DA, UK; HZPC Research B.V. HZPC, Edisonweg 5, 8501 XG Joure, Netherlands; College of Plant Protection, China Agricultural University, Haidian District, Beijing, 100083, China; The James Hutton Institute, Errol Road, Invergowrie, Dundee, DD2 5DA, UK; Division of Plant Sciences at the Hutton, The University of Dundee, Errol Road, Invergowrie, Dundee, DD2 5DA, UK; The James Hutton Institute, Errol Road, Invergowrie, Dundee, DD2 5DA, UK; College of Plant Protection, China Agricultural University, Haidian District, Beijing, 100083, China

## Abstract

Potato is the third most important food crop in the world. Diverse pathogens threaten sustainable crop production but can be controlled, in many cases, through the deployment of disease resistance genes belonging to the family of nucleotide-binding, leucine-rich-repeat (NLR) genes. To identify effective disease resistance genes in established varieties, we have successfully established SMRT–AgRenSeq in tetraploid potatoes and have further enhanced the methodology by including dRenSeq in an approach that we term SMR–AgRenSeq-d. The inclusion of dRenSeq enables the filtering of candidates after the association analysis by establishing a presence/absence matrix across resistant and susceptible varieties that is translated into an F1 score. Using a SMRT–RenSeq-based sequence representation of the NLRome from the cultivar Innovator, SMRT–AgRenSeq-d analyses reliably identified the late blight resistance benchmark genes *Rpi-R1*, *Rpi-R2-like*, *Rpi-R3a*, and *Rpi-R3b* in a panel of 117 varieties with variable phenotype penetrations. All benchmark genes were identified with an F1 score of 1, which indicates absolute linkage in the panel. This method also identified nine strong candidates for *Gpa5* that controls the potato cyst nematode (PCN) species *Globodera pallida* (pathotypes Pa2/3). Assuming that NLRs are involved in controlling many types of resistances, SMRT–AgRenSeq-d can readily be applied to diverse crops and pathogen systems.

## Introduction

Potato is the third most important global food crop and is both nutritionally and economically valuable [1]. Potato is a staple of many diets around the world, and diseases can thus have a significant impact on crop production and food security. Diverse and phylogenetically unrelated pathogens including oomycetes, fungi, bacteria, viruses, nematodes, and insects can cause significant crop losses in potato [2].

The potato cyst nematode (PCN) species *Globodera rostochiensis* and *Globodera pallida* are economically important pathogens of potato and are present in most potato-growing regions of the world [3]. PCN is difficult to eradicate once established as cysts can survive for over 20 years in the soil, rendering even longer crop rotations insufficient for clearing land [4, 5]. Thus, the impact of PCN goes beyond the immediate yield losses, which are estimated to be around 9% in susceptible potatoes at low levels of nematode infestation but can be significantly more when nematode pressure is high. Further, contaminated land is rendered unsuitable for high-health potato seed production which in turn can impact on the crop supply chain [4, 6].

The only cloned disease resistance genes effective against PCN are *Gpa2* and *Gro1–4* which encode for canonical nucleotide-binding, leucine-rich-repeat (NLR) genes [7, 8]. In current potato cultivars, the most effective resistance gene used to control *G. pallida* is *Gpa5*, which has been introduced from the wild Solanaceae species *Solanum vernei* [9]. The resistance has been mapped to potato chromosome 5 [9, 10] and a haplotype-specific PCR marker, HC, was developed which corresponds in its position to an NLR-rich locus on chromosome 5 [11]. This locus is also associated with the late blight resistance gene *Rpi-R1*, amongst other functional NLRs such as *Grp1* and *H2* [3, 12]. Intriguingly, Rouppe van der Voort *et al.* [9] and an independent study aimed at mapping a *S. vernei* source of resistance against *G. pallida*, independently revealed a further QTL, *Gpa6*, on chromosome 9 that complements the major effect mapped to chromosome 5 [13, 14].

To specifically study potato NLRs, RenSeq was developed as a target enrichment tool following the annotation of NLRs in the reference genome DM1–3516 R44 (DM) [15–17]. In combination with PacBio-based sequencing of long genomic DNA fragments, described as SMRT–RenSeq, the technology enables the representation of entire plant NLRomes [18, 19]. A diagnostic version of RenSeq, dRenSeq, has been used to ascertain the presence/absence of known NLRs in wild species or cultivars. Thus, dRenSeq aids the identification of novel resistances in germplasm collections as well as the deployment and pyramiding of complementary resistances in cultivars [20, 21].

RenSeq has also been adapted for association genetics (AgRenSeq) which takes advantage of independent recombination events that occur during meiosis. AgRenSeq, which determines the potential of candidate NLRs being responsible for resistance traits, uses *k*-mer-based association and has initially been implemented in *Aegilops tauschii,* a wild progenitor of the D subgenome of hexaploid bread wheat [22]. AgRenSeq has also been performed with PacBio HiFi reads to identify candidate resistance genes in rye controlling *Puccinia recondita* f. sp. *secalis* [23]*.* This approach was termed SMRT–AgRenSeq.

Here we developed SMRT–AgRenSeq-d in potatoes to identify candidate NLRs that are associated with the *Gpa5* resistance linked to the HC marker. To study *Gpa5*, we selected the tetraploid potato cultivar Innovator as a reference, since this cultivar is known to exhibit high levels of *G. pallida* resistance based on the presence of *Gpa5* [3]. As previously shown, Innovator also contains four known functional NLRs effective against *Phytophthora infestans*: *Rpi-R1*, *Rpi-R2-like*, *Rpi-R3a*, and a variant of *Rpi-R3b* [20]. These four NLRs reside in three clusters of different sizes and thus function as ideal benchmark genes to test the accuracy of the NLR assembly and subsequent association studies. Using the RenSeq dataset from 117 potato lines, SMRT–AgRenSeq-d successfully identified all full-length late blight benchmark genes and facilitated the identification of candidate NLRs for *Gpa5*. This highlights the versatility of the RenSeq-based association genetics approach in studying disease resistance against phylogenetically unrelated pathogens and underscores the additional value this method can provide. Further, incorporating dRenSeq analysis allows for candidate validation and the identification of NLRs that are 100% linked to the resistance phenotype. In this study, we express recombination rates using an F1 score, which informs the order of paralogs within the genetic interval and narrows down the candidates to those with an F1 score of 1. Thus, we suggest that this technology is well- suited for identifying resistances against a wide range of pathogens controlled by functional NLRs and can be applied to different crops [24].

## Results and discussion

SMRT-AgRenSeq-d encompasses a representation of the NLRs from a plant with the desired resistance, a panel of plants with contrasting phenotypes to ascertain the *k*-mer-based association, and dRenSeq-based assessment of candidates. The benchmark genes *Rpi-R1*, *Rpi-R2-like*, *Rpi-R3a*, and *Rpi-R3b* in Innovator were used to provide a quality check throughout the analysis.

### *De-novo* assembly of Illumina-based RenSeq reads fails to represent benchmark NLRs

To assess the accuracy of the NLR representation from Illumina sequences, we used RenSeq reads generated from the potato cultivar Innovator [20]. The integrity of the four benchmark genes was assessed in the assembled contigs (Table 1). Only *Rpi-R1* was accurately and completely represented in the contig scf7180000044808. In contrast, *Rpi-R2-like*, and the *Rpi-R3b* variant were partially and incorrectly represented in contigs scf7180000045065 and scf7180000045838, respectively. Representation of *Rpi-R3a* was split over two contigs, scf7180000044942 and scf7180000045673.

**Table 1:** Representation of four benchmark genes in the potato cultivar Innovator based on Illumina and PacBio RenSeq read assemblies

**Reference Gene**	**Representative Sequence**	**Identity (%)**	**Mismatch**	**Gap**	**Reference gene start**	**Reference gene end**	**Gene Length**	**Coverage of Gene (%)**	**Coverage of HSP** ^**^ **(%)**
**Illumina reference contigs**									
*Rpi-R1*	scf7180000044808	100	0	0	1	4103	4103	100	100
*Rpi-R2-like*	scf7180000045065	92.635	170	4	1	2539	2545	99	99
*Rpi-R3a*	scf7180000044942	99.881	3	0	1	2531	3850	66	66
*Rpi-R3a*	scf7180000045673	99.937	1	0	2254	3850	3850	41	41
*Rpi-R3b^G1696/G3111^*	scf7180000045838	99.958	1	0	698	3073	3853	62	62
**PacBio reference contigs**									
*Rpi-R1*	tig00000086	100	0	0	1	4103	4103	100	100
*Rpi-R2-like*	tig00001243	100	0	0	1	2545	2545	100	100
*Rpi-R3a*	tig00000180	100	0	0	1	3850	3850	100	100
*Rpi-R3b^G1696/G3111^*	tig00000301	99.974	1	0	1	3853	3853	100	100

a A High-scoring Segment Pair (HSP) is a local alignment with no gaps that achieves one of the highest alignment scores in a given search.

### PacBio HiFi-based assembly of RenSeq reads generates a high-quality NLRome representation

The combination of RenSeq with single-molecule real-time (SMRT) sequencing (SMRT-RenSeq) has overcome the limitations of Illumina-based assemblies in the cloning of NLRs in wild diploid potato species [19]. The assembly of HiFi RenSeq reads from Innovator yielded 4,885 contigs with an average length of 11,279 bp. Amongst those, 1,760 contigs contained a total of 2,418 predicted NLRs (Supplementary Table S1).

In this assembly, the benchmark genes *Rpi-R1*, *Rpi-R2-like*, and *Rpi-R3a* were represented completely and accurately in a single contig each (Table 1). A comparison between the previously identified variant of *Rpi-R3b* in Innovator, *Rpi-R3b^G1696/G3111^*, with the well supported PacBio contig tig00000301 revealed 100% coverage of this gene and 99.974% identity to *Rpi-R3b^G1696/G3111^* with only one mismatch at position C918. We referred to this variant as *Rpi-R3b^C918/G1696/G3111^.* Illumina RenSeq reads from Innovator and other cultivars, except for Brodie, could not distinguish *Rpi-R3b^G1696/G3111^* from *Rpi-R3b^C918/G1696/G3111^* (Supplementary Table S2). This suggests that highly similar sequences obscure the additional sequence polymorphism, or that some cultivars contain both variants.

These results independently confirm and quantify the advantages of SMRT–RenSeq in accurately representing the complex NLR gene family. This is particularly valuable as NLR genes often share highly similar sequences due to gene duplications. Illumina reads, while highly accurate, are often too short to distinguish homologous sequences accurately, leading to incorrect assembly of related genes. Consequently, Illumina-based assemblies are inadequate for representing more complex NLR gene families [18, 23, 25]. In addition, these contigs, which are larger in size compared to the described Illumina-derived contigs, can contain multiple NLRs and their flanking regions on single contigs, which aids functional analyses such as expression of candidates with their native regulatory elements.

**Figure 1:**
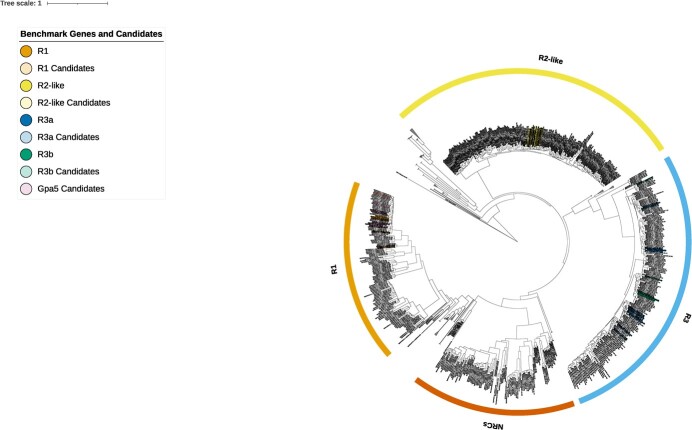
Phylogenetic tree displaying NB domains of Innovator NLRs following assembly of SMRT-RenSeq reads. Expanded are the clades containing *NRCs*, *Rpi-R1*, *Rpi-R2-like*, *Rpi-R3a*, and *Rpi-R3b*. Collapsed clades either consist of automatic predictions from NLR-Annotator or of NLR clades not investigated in this study. Highlighted are the identified benchmark genes and candidates from each association with F1 = 1. The NB containing proteins APAF1 from *Homo sapiens* and CED4 from *Caenorhabditis elegans* act as outgroups. The tree scale indicates substitutions/site.

A phylogenetic tree was constructed from the SMRT-RenSeq derived NLRs using the NB domains of predicted NLRs from Innovator (Figure 1; Supplementary Figure S1)*.* The resulting tree shows clear groupings of NLRs into numerous distinct clades and emphasizes the scale of duplications that have occurred in tetraploid potatoes. In addition to highlighting the NLRs related to *Rpi-R1*, *Rpi-R2-like*, *Rpi-R3a*, and *Rpi-R3b*, a clear NLR Required for Cell death (NRC) clade [26] is apparent (Figure 1; Supplementary Figure S1).

### Development of an association panel

The cultivar Innovator was released in 1999 and has since been extensively used in breeding programs and is attributed as a direct parent to over 25 named varieties [27] (Supplementary Table S3). To study *Gpa5* and to take advantage of the wide genetic reach of Innovator, we developed an association panel of 117 potato accessions with contrasting resistance/susceptible phenotypes against *G. pallida*, associated with the presence/absence of *Gpa5* (Supplementary Table S2).

We assessed the same panel for the sequence representation of the late blight resistance genes *Rpi-R1*, *Rpi-R2-like*, *Rpi-R3a*, and *Rpi-R3b* through dRenSeq, as they have been identified in Innovator previously [20]. Informed by the natural diversity of *Rpi-R2-like*, which in the cultivar Pentland Dell has 97.4% dRenSeq coverage [20], we set a minimum cutoff value of more than 97% sequence representation for a gene to be classified as ‘present’. This was converted into a presence/absence matrix for the four genes, which form the benchmarks for this study, across the association panel (Supplementary Table S2).

### SMRT-AgRenSeq-d development for *Rpi-R3a* as a proof of concept

**Figure 2:**
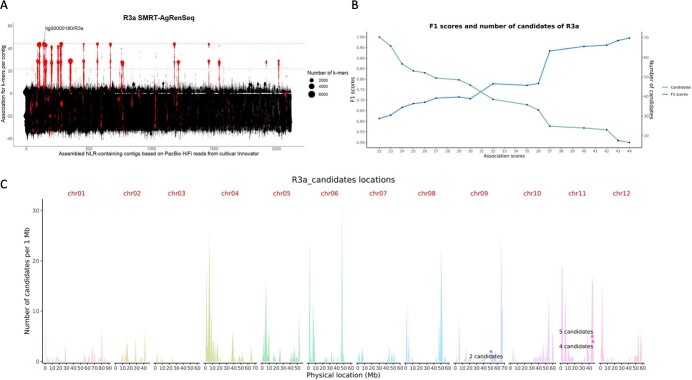
SMRT-AgRenSeq-d of *Rpi-R3a*. (a) Identification of NLR containing contigs in the reference cultivar Innovator with association to *Rpi-R3a*. Columns on the x-axis represents NLR containing contigs, with dots indicating mapped *k*-mers and their association score to *Rpi-R3a* on the y-axis. Dashed lines highlight highest association score (44, upper line) and chosen threshold (22, lower line). (b) F1 scores and number of candidate NLRs for each association score group. (c) The predicted positions of *Rpi-R3a* candidates with F1 = 1 are shown (dots) in relation to all NLRs from the reference DM v6.1 mapped over all 12 chromosomes (peaks).

Since potato is an autotetraploid crop with complex tetrasomic inheritance patterns, we assessed first the suitability of SMRT-AgRenSeq-d on the benchmark genes *Rpi-R1, Rpi-R2-like, Rpi-R3a*, and *Rpi-R3b* (Figure 2; Supplementary Figures S2-S4).

The SMRT-AgRenSeq-d study for *Rpi-R3a* yielded a maximum positive association score of 44 (Figure 2a). To systematically assess the resulting candidates and to be as inclusive as possible in the analysis, we selected half of the maximum association score as an initial cutoff for candidate selection. At this lower association score of 22, 46 contigs were identified that contain 73 candidate NLRs. The sequences of these 73 predicted NLRs were analysed by dRenSeq to independently validate their prevalence in the 44 clones that contain the reference sequence of *Rpi-R3a* and their absence in the 73 potato clones identified as missing *Rpi-R3a* (Supplementary Table S4). Based on this graphical genotyping depicting the presence/absence of candidate genes in the respective pools, an F1 score [28] was determined. An F1 score of 1 indicates the presence of the candidate gene in all clones from the positive panel and the consistent absence in all negative clones. Importantly, the *Rpi-R3a* containing contig tig00000180 (Table 1) was amongst these highly positively associated candidates (Supplementary Table S4).

A score of less than 1 therefore indicates false positives (e.g. candidates that are not present in all clones from the positive set) and/or false negatives (e.g. candidates that are present in the negative set). This conceptually equates to identifying recombination events in potato clones that have, for example, lost a specific candidate. Thus, the F1 score, which is determined through the dRenSeq analysis, complements the association score by confirming and further prioritising candidates. Interestingly, candidates that yielded an F1 score of less than 0.7, some of which had a relatively high association score of 37, displayed unsystematic presence/absence patterns that are inconsistent with robust association.

The analysis of candidates with an F1 score of larger than 0.7 but smaller than 1 highlighted examples of potential recombination and false positives, including two NLRs with an association score of 44 from contig tig00000196 in the cultivar Cammeo that does not contain *Rpi-R3a* (Supplementary Table S4). Consequently, their F1 scores were lower than 1 at 0.9887. *Rpi-R3b* was also present in the *Rpi-R3a* candidate gene list, on contig tig00000301 (Table 1, Supplementary Table S4). This is consistent with the finding that all *Rpi-R3a* containing clones also contained *Rpi-R3b* (Supplementary Table S2). However, as other potato clones also contain *Rpi-R3b*, yet do not contain *Rpi-R3a*, the F1 score for this contig was again less than 1. Consistent with this analysis, we found that the number of candidates is inversely related to the F1 score (Figure 2b).

Only 11 putative NLRs had an F1 score of 1 and, as mentioned above, include contig tig00000180 that contains the full-length *Rpi-R3a* gene sequence alongside a closely related gene. We surmise that these 11 genes are physically linked and likely represent paralogous sequences, as alleles would have been genetically randomly distributed owing to the tetrasomic inheritance patterns of potato. Further, when mapped to the DM reference genome, nine out of the 11 candidates resided in the predicted *Rpi-R3* locus on chromosome 11 (Figure 2c). The remaining two genes had their best BLASTn hit on DM chromosome 9 which contains sequences that belong to the *Rpi-R3* family as demonstrated by Jupe *et al.* [16]. Consistent with this, all 11 candidates reside in the *Rpi-R3a/R3b* clade of the phylogenetic tree (Figure 1).

### SMRT-AgRenSeq-d validation for benchmark genes *Rpi-R3b*, *Rpi-R2*-like, and *Rpi-R1*

Using the same SMRT-AgRenSeq-d approach for the benchmark genes *Rpi-R3b*, *Rpi-R2-like*, and *Rpi-R1* independently validated our methodology and successfully identified the functional genes amongst the positively associated contigs and specifically amongst those yielding an F1 score of 1. Thus, the integration of dRenSeq to validate the association of NLRs and to determine the F1 score is a valuable step in reducing the number of candidates rapidly.

Following the SMRT-AgRenSeq-d analyses for *Rpi-R3b*, 83 NLR containing contigs with 114 NLRs were identified with a minimum association score of 30 (Supplementary Figure S2). When filtered for an F1 score of larger than 0.7, 21 contigs that contained 31 NLRs were identified with an association score of between 56 and 60 (Supplementary Table S5). These include tig00000194 and tig00000301 of which the latter contains *Rpi-R3b* (Table 1). Furthermore, all genes map to the *Rpi-R3* locus on potato chromosome 11 (Supplementary Figure S2c) and reside in the phylogenetic clade associated with *Rpi-R3b* (Figure 1).

Representation of the *Rpi-R2* gene family in the association set was the lowest of all benchmark genes. Despite less than 10% penetration of the phenotype in the panel (10 of 117; Supplementary Table S2), SMRT-AgRenSeq-d identified ten candidate NLRs with an F1 score of 1 which include the *Rpi-R2-like* containing contig tig00001243 (Supplementary Figure S3; Supplementary Table S6, Table 1). These candidates all map to the *Rpi-R2* locus on potato chromosome 4 (Supplementary Figure S3c) and are members of the *Rpi-R2* clade in the phylogenetic tree (Figure 1).

The validation of SMRT-AgRenSeq-d on the *Rpi-R1* benchmark gene yielded the lowest number of associated contigs (Supplementary Figure S4), which is most likely a reflection of the smaller size of the *Rpi-R1* gene cluster compared to *Rpi-R2* and *Rpi-R3a/R3b*. Indeed, in DM, *Rpi-R1* is one of the smallest clusters of NLRs [16]. At a minimum association score of 17, 16 NLR containing contigs containing 20 NLRs were identified (Supplementary Figure S4). After filtering for an F1 score of larger than 0.7, 11 contigs had association scores between 22 and 34 and those encode for 13 NLRs (Supplementary Table S7). The *Rpi-R1* containing contig, tig00000086 (Table 1) was successfully identified with an F1 score of 1.

### SMRT-AgRenSeq-d identified nine candidates for Gpa5

Following the development and independent validation of SMRT-AgRenSeq-d on all four benchmark genes, the approach was applied to identify candidates for the resistance gene *Gpa5*. The association panel of 117 potatoes, as described above, includes 23 highly resistant clones with a *G. pallida* resistance score of between 7 and 9 and which tested positive for the HC marker [11], alongside 94 susceptible clones with a resistance score of 1–3 (Supplementary Table S2).

**Figure 3:**
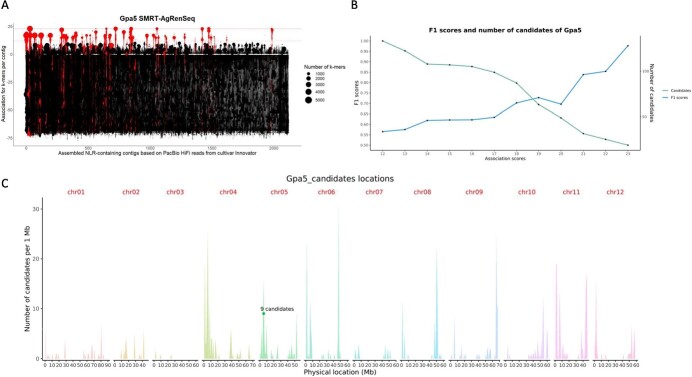
SMRT-AgRenSeq-d of *Gpa5*. (a) Identification of NLR containing contigs in the reference cultivar Innovator with association to *Gpa5*. Columns on the x-axis represent contigs with signals of NLRs, with dots indicating mapped *k*-mers and their association score to *Gpa5* on the y-axis. Dashed lines highlight highest association score (23, upper line) and chosen threshold (12, lower line). (b) F1 score and number of candidate NLRs for each association score group. (c) The predicted positions of *Gpa5* candidates with F1 = 1 are shown (dots) in relation to all NLRs from the reference DM v6.1 mapped over all 12 chromosomes (peaks).

Conducting SMRT-AgRenSeq-d on this panel of highly resistant and highly susceptible potatoes identified 78 NLR-containing contigs with 139 predicted NLRs and with a minimum association score of 12 (Figure 3a).

Following dRenSeq analysis, nine NLRs with an F1 value of 1 were identified and were considered strong candidates for the resistance (Supplementary Table S8). Consistent with Sattarzadeh *et al.* [11] and van Eck *et al.* [10], these nine candidates map to the *Rpi-R1* locus on potato Chromosome 5 (Figure 3c; Supplementary Table S8) and phylogenetically cluster with the *Rpi-R1* gene family (Figure 1).

Genetically, this study provides evidence that *Gpa5* is in repulsion to *Rpi-R1* as the *Rpi-R1* containing contig tig00000086 (Table 1) was not associated with the nematode resistance (Supplementary Table S8). This is further supported by the presence/absence of *Rpi-R1* in the association panel, where more than 60% (14 out of 23) of clones that contained *Gpa5* did not contain *Rpi-R1* (Supplementary Table S2). Thus, we clearly demonstrate that *Rpi-R1* and the *Gpa5* candidates are allelic genes that recombine independently of each other.

To further validate these nine potential candidates in providing resistance, we independently assessed the presence/absence of these candidate genes in three additional resistant potato cultivars (Tiger, Novano, and Karelia) and five breeding clones that tested positive for the HC marker (Supplementary Table S9). As a negative control, we included the cultivar Vales Everest which displayed intermediate to high resistance against *G. pallida* Pa2/3. However, this cultivar utilises the *H3* resistance instead of *Gpa5* [29]. Except for Vales Everest, all candidates with an F1 score of 1 were consistently identified in this set including the lines with intermediate phenotypes.

## Conclusion

In conclusion, we have further developed SMRT-AgRenSeq by including a dRenSeq analysis that expedites the process of identifying candidates that are linked to the resistance phenotype. Validation of this linkage is underway through functional analyses. The F1 score that can be determined for all candidates in this combined approach is an informative measure of linkage and proved more informative than, for example, the association score alone. The resulting method, SMRT-AgRenSeq-d, successfully identified the benchmark genes *Rpi-R1*, *Rpi-R2-like*, *Rpi-R3a*, and *Rpi-R3b* with the highest possible F1 score of 1 in the association panel. Importantly, SMRT-AgRenSeq-d correctly identified those genes even though their penetration in the panel varied between 8.5% for *Rpi-R2-like*, 29.3% for *Rpi-R1*, 37.6% for *Rpi-R3a*, and 51.3% for *Rpi-R3b*. When applied to *Gpa5*, which is currently the most effective and widespread resistance against *G. pallida* used in potato crop protection, SMRT-AgRenSeq-d identified strong candidate NLRs. All *Gpa5* candidates mapped to the previously established locus on potato chromosome 5 and were shown to be phylogenetically related to the late blight resistance gene *Rpi-R1*.

Considering the wide use of RenSeq in diverse plant species ranging from *Arabidopsis thaliana* [18] and* Haynaldia villosa* [25] to crops such as potato, tomato [30], and rye [23], we propose that this refined method is suitable for studying NLR-based resistances in various crops against a wide range of pathogens. Indeed, depending on the availability of complementary phenotypic data, existing RenSeq datasets can be leveraged to study resistances to unrelated pathogens. This versatility is exemplified in our study, where we applied RenSeq to identify candidate NLRs for *Gpa5* effective against PCN, whilst using the identification of three late blight NLRs as a proof of concept.

## Materials and methods

### Source of plant and pathogen material

*G. pallida* cysts that were used in the phenotyping test were of the Pa2/3 population known as ‘Lindley’ [31]. The population was multiplied on the susceptible cultivar Desiree in glasshouses in 2019. All potato accessions were grown in the glasshouse. In some cases, lyophilised DNA was received from collaborators.

### Extraction of DNA

Bulk DNA was extracted from leaves of all potato accessions using the Qiagen DNEasy Plant Mini Kit (Qiagen, Hilden, Germany) following the manufacturer's instructions. DNA for HiFi sequencing was extracted using the Wizard® HMW DNA Extraction Kit (Promega, Madison, WI, USA), following the manufacturer's instructions.

### RenSeq

The RenSeq bait library was as described in Armstrong *et al.* [20]. Enrichment sequencing was performed by Arbor Biosciences (Ann Arbor, MI, USA) using the Novaseq 6000 platform for Illumina reads and the PacBio Sequel II platform for HiFi reads.

### Assembly

Raw Illumina reads were mapped to the DM v6.1 genome assembly [32] with BWA-mem v0.7.17-r1188 [33] using default parameters. Picard CollectInsertSizeMetrics v2.25.1 [34] was used to calculate the mean and standard deviation of the read insert size. This was used to inform the MASURCA [35] v3.4.2 assembly with PE = pe 342 97 and CLOSE_GAPS = 1 as non-default parameters. Assembly of HiFi reads was conducted using HiCanu with the SMRT–RenSeq Assembly workflow within HISS v2.0.0 [36, 37].

### Identification of known resistance genes

The CDS sequences of *Rpi-R1*, *Rpi-R2-like*, *Rpi-R3a*, and *Rpi-R3b* [38–41] were searched for in both sets of assembled contigs using BLASTN v2.11.0 [42, 43] with an e-value of 1e^−5^.

### Phylogenetics

NLR sequences from the SMRT-RenSeq assemblies were predicted using NLR-Annotator [44] and nucleotide binding (NB) domains were identified with Interproscan version 5.54-87.0 [45, 46] with goterms, iprlookup, and pathways options enabled. The output XML file was parsed with a custom python script to extract the amino acid sequences of the NB domains (IPR002182). Reference NRC amino acid sequences were retrieved from Adachi *et al.* [47], submitted to Interproscan and the output tsv file was parsed with a different custom python script. The BED files produced by these scripts were used to extract FASTA sequences with the bedtools getfasta function (version 2.30.0) [48]. The known NLR and NRC gene NB domain sequences were aligned with Clustal Omega version 1.2.4 [49] with 10 iterations. Following this, the NB domains of the automatically predicted NLRs were added to the alignment with Clustal Omega using the previous alignment as a profile, again with 10 iterations.

Tree construction was performed in R v4.1.3 [50] using the libraries ape v5.6-2 [51] and phangorn v2.9.0 [52]. Sites and sequences with 85% missing data, or more, were removed from the alignment. Following this, duplicated sequences were removed from the alignment. A phylogeny based on maximum likelihood was inferred from the final alignment. The Bayesian information criteria (BIC) obtained from the ‘modelTest’ function of phangorn suggested a JTT+G model. The tree was bootstrapped with 1000 replicates and its topology was optimized using nearest neighbour interchanges (NNI). The phylogeny produced was then rooted to the outgroup sequences from *Homo sapiens* and *Caenorhabditis elegans*. Clades were assigned with PhyloPart 2.1 [53] with a percentile threshold of 0.05. The figure was created in iToL [54], details on how to replicate the figure and text files for modifying the view are described in a markdown file.

### Diversity panel formation and additional phenotyping

The resistance or susceptibility of clones within the association panel was determined by retrieving results from potato cultivar databases and/or through replicated PCN tests. Only resistant clones that also tested positive for the HC marker [11] were considered to ensure that the source of the resistance is consistent (Supplementary Table S3; Supplementary Table S9). The phenotyping experiment was based on the method published by [55]. Briefly, tubers of breeding clones and six controls (Desiree, Maris Piper, Vales Everest, Lady Balfour, Innovator and King Russet) were planted in canisters (one tuber per canister) with four replicates. All canisters were placed into four trays, each containing one replicate of all breeding clones and controls in a randomized design. Canisters were inoculated with approximately 10–15 cysts. After inoculation, canisters were stored in the dark at 18°C for 7 weeks. After this time, the number of females observed on the root balls were counted, and the phenotypic scores were recorded. In these accessions and breeding clones, 117 lines whose scores were between 1 and 3 (susceptible accessions) (n = 94) and 7–9 (resistant accessions) (n = 23) were chosen for the diversity panel for *Gpa5*.

To determine the presence/absence of the late blight resistance genes *Rpi-R1*, *Rpi-R2-like*, *Rpi-R3a*, and *Rpi-R3b* that are present in Innovator, dRenSeq was performed as described previously by Armstrong *et al.* [20]. Based on the known diversity within the *Rpi-R2* family [20], we set a minimum sequence coverage threshold of 97% for a gene to be classified as present.

### SMRT-AgRenSeq-d

SMRT-AgRenSeq-d was performed using the AgRenSeq and dRenSeq workflows in HISS v2.0.0 [36]. AgRenSeq followed the workflow initially described for *A. tauschii* [22]. Briefly, the Illumina reads of the previously described panel of samples were assessed for the presence and absence of *k*-mers with a *k* value of 51 and arranged into a presence/absence matrix. This matrix was then used alongside phenotypic information to perform the association and the identities of contigs containing *k*-mers highly associated with the phenotype were reported.

An initial threshold of 50% of the highest association score was used for selecting candidate contigs and the NLR coordinates were determined with NLR-Annotator [44] (*Rpi-R1*: 17; *Rpi-R2-like*: 5; *Rpi-R3a*: 22; *Rpi-R3b*: 30; *Gpa5*: 12). These selected contigs were used for dRenSeq. This followed the pipeline previously described [20, 56] whilst specifically reporting on NLR regions linked to bait coverage. Briefly, the Illumina reads of the panel were mapped to the CDS regions of candidate genes. This mapping was then filtered to contain only reads with zero mismatches within regions also represented by baits with at least 90% sequence identity. The resulting coverage of the candidates was reported.

### Refining of candidates

Candidates identified by SMRT-AgRenSeq-d were further refined through the generation of precision and recall values and calculation of the F1 score of the candidates [28]. The following formula was applied:

$$ F1=\frac{2\times PPV\times TPR}{PPV+ TPR} $$

PPV is the positive predictive value or precision, calculated by the following formula:

$$ PPV=\frac{TP}{TP+ FP} $$

TPR is the true positive rate or sensitivity/recall, calculated by the following formula:

$$ TPR=\frac{TP}{TP+ FN} $$

Here, TP represents true positive cases, i.e. varieties/clones that are phenotypically resistant and have complete coverage of the candidate in dRenSeq (within the parameters specified above). FP (false positive) refers to varieties that are phenotypically susceptible but still had complete coverage in dRenSeq. Conversely, TN (true negative) refers to varieties that are phenotypically susceptible and had only partial coverage in dRenSeq, whereas FN (false negative) refers to phenotypically resistant varieties with partial coverage in dRenSeq. The individual F1 scores were calculated for every candidate.

## Supplementary Material

Web_Material_uhad211

## Data Availability

BioProjects: ERP141787—Illumina Reads/contigs. ERP141789—HiFi Reads/contigs. Code: https://github.com/TMAdams/gpa5_smrt_agrenseq_paper
