# Supplementary material for: SMRT–AgRenSeq-d in potato (Solanum tuberosum) as a method to identify candidates for the nematode resistance Gpa5
Source: Hortic Res. 2023 Oct 17;10(11):uhad211. doi: 10.1093/hr/uhad211 (PMC10681002; doi:10.1093/hr/uhad211)
Supplement: Web_Material_uhad211 [file web_material_uhad211.zip › Supplementary_Figure_1.pdf]

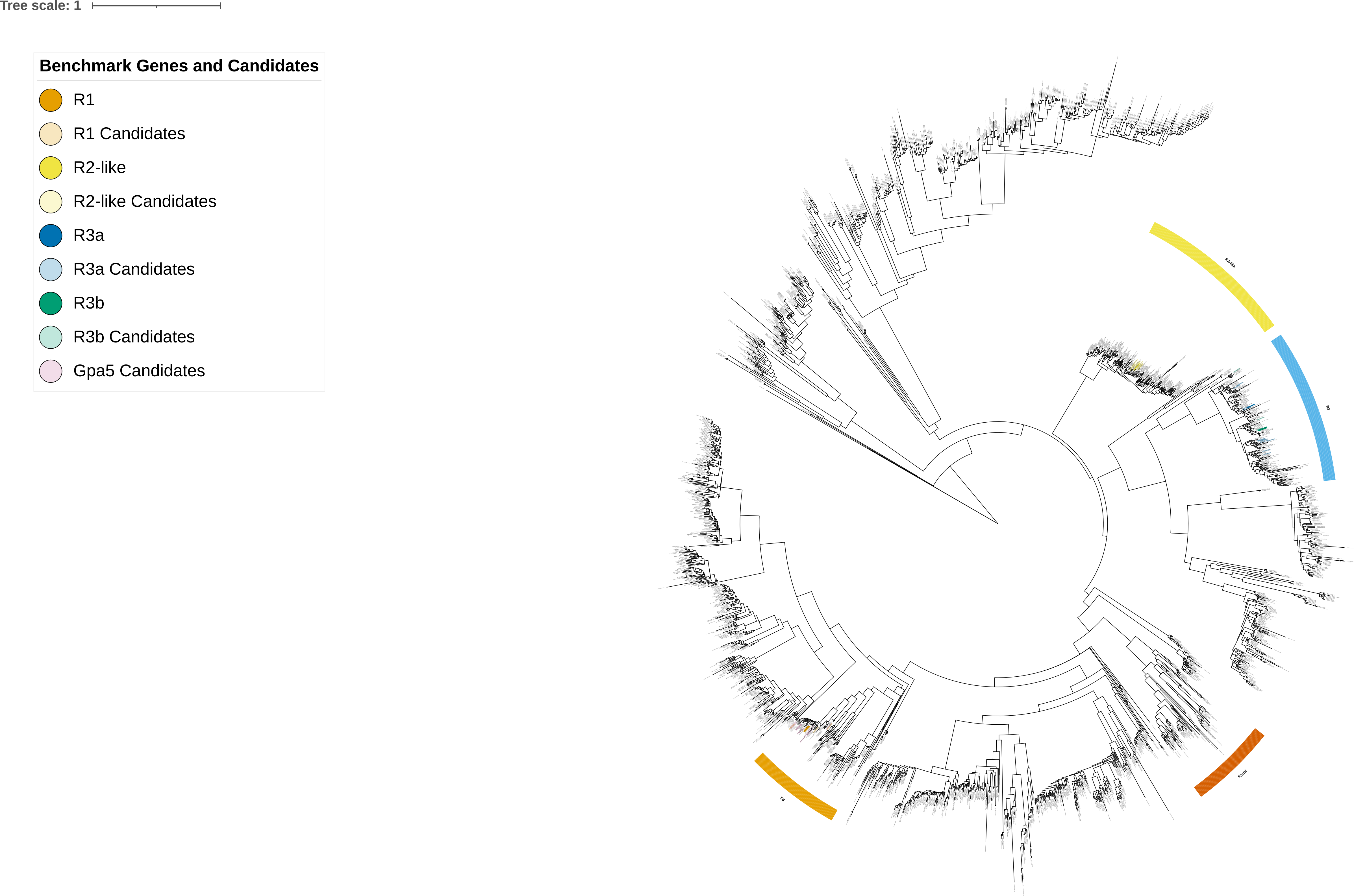
Tree scale: 1
Benchmark Genes and Candidates
R1
R1 Candidates
R2-like
R2-like Candidates
R3a
R3a Candidates
R3b
R3b Candidates
Gpa5 Candidates
