# Supplementary figures and images for: SMRT–AgRenSeq-d in potato (Solanum tuberosum) as a method to identify candidates for the nematode resistance Gpa5
Source: Hortic Res. 2023 Oct 17;10(11):uhad211. doi: 10.1093/hr/uhad211 (PMC10681002; doi:10.1093/hr/uhad211)

A

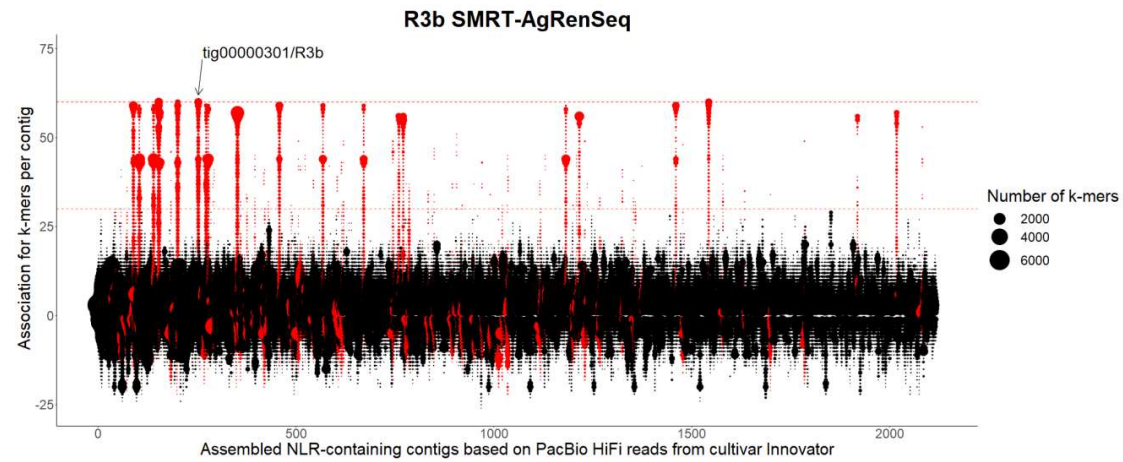

B

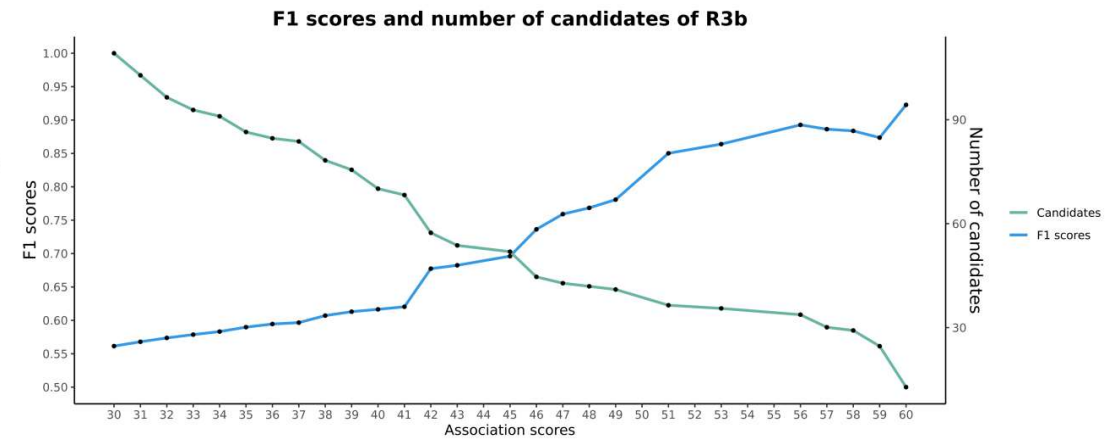

C

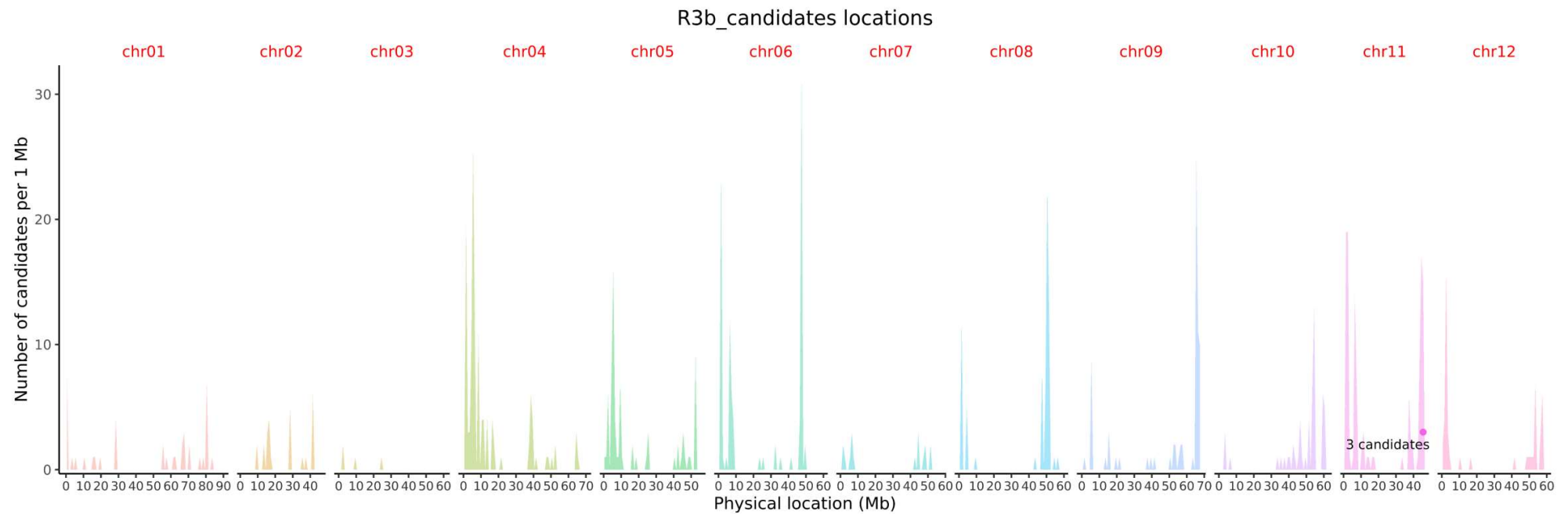

Supplement: Web_Material_uhad211 [file web_material_uhad211.zip › SF2_Supplementary Figure 2 R3b.pdf]

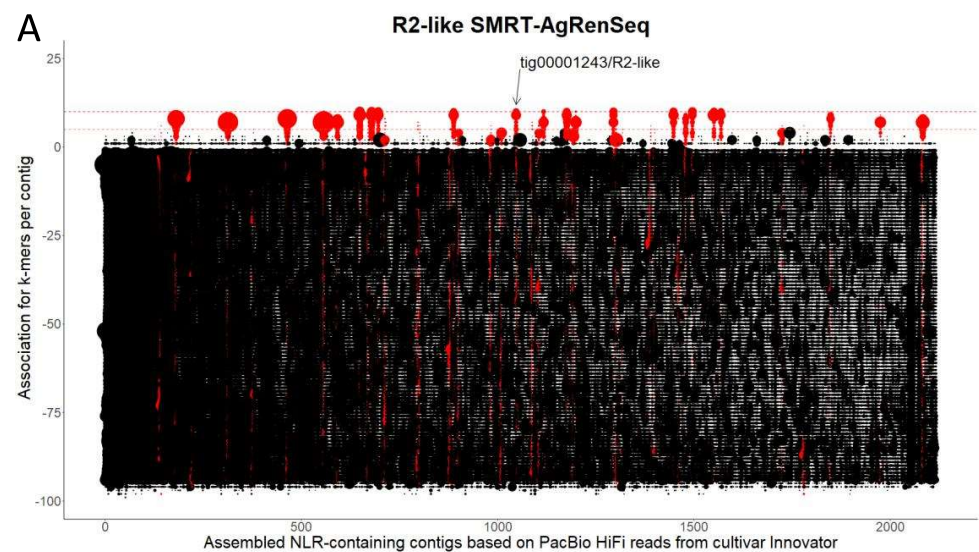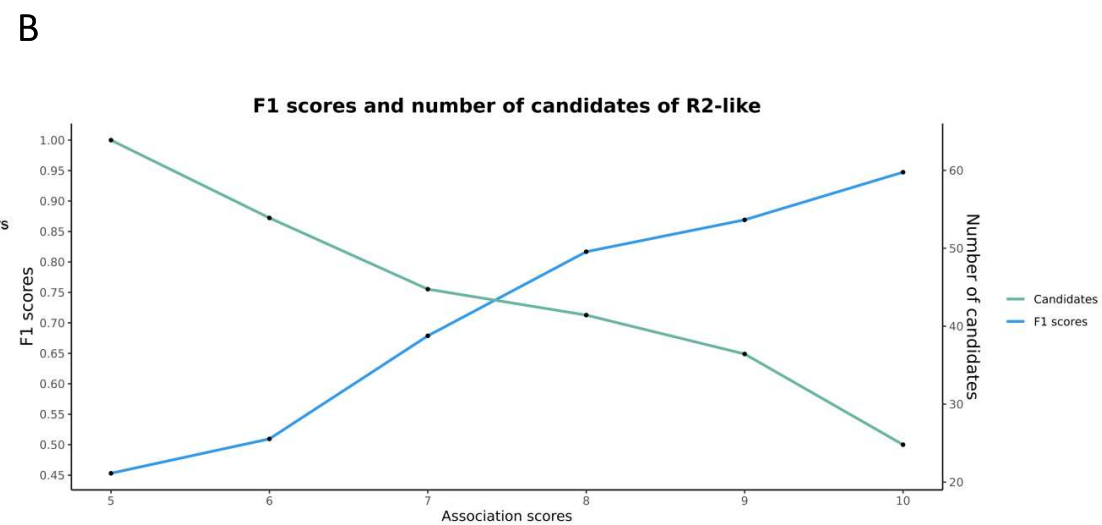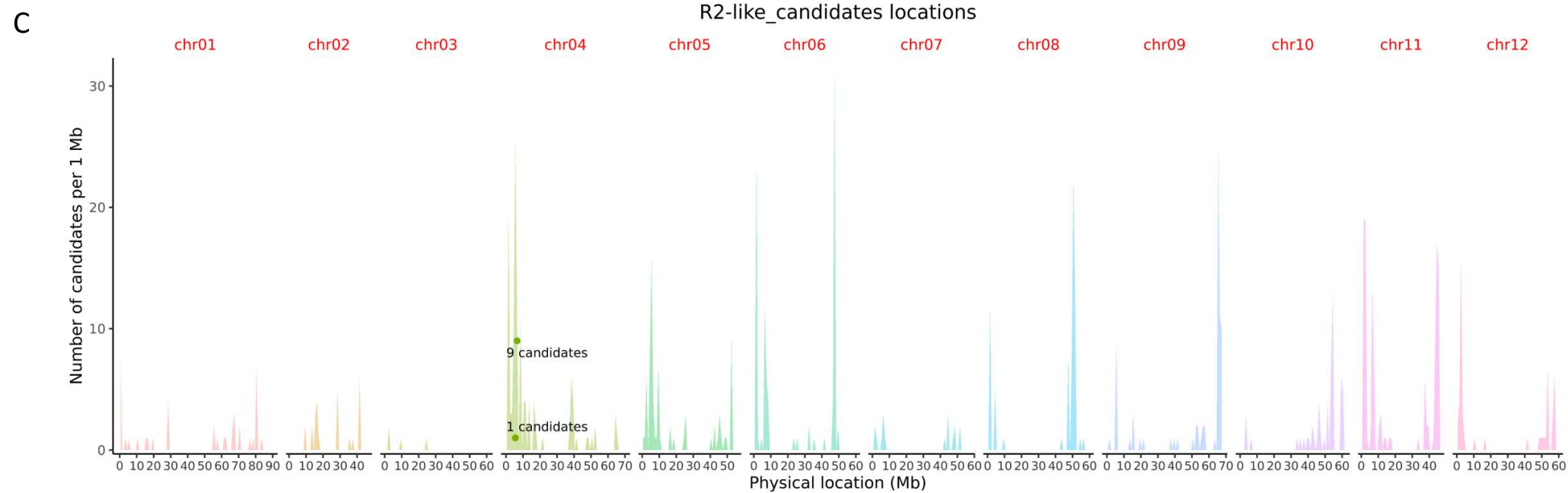

Supplement: Web_Material_uhad211 [file web_material_uhad211.zip › SF3_Supplementary Figure 3 R2-like.pdf]

A

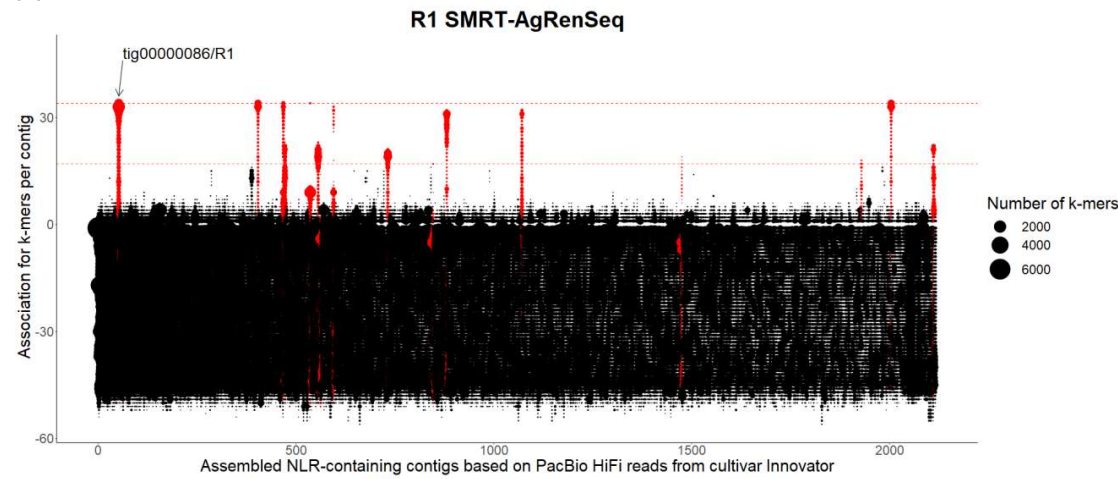

B

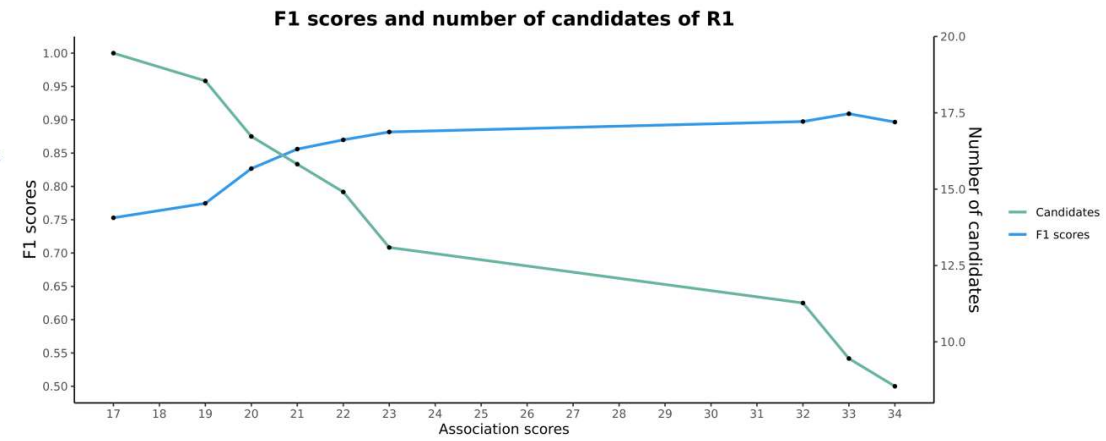

C

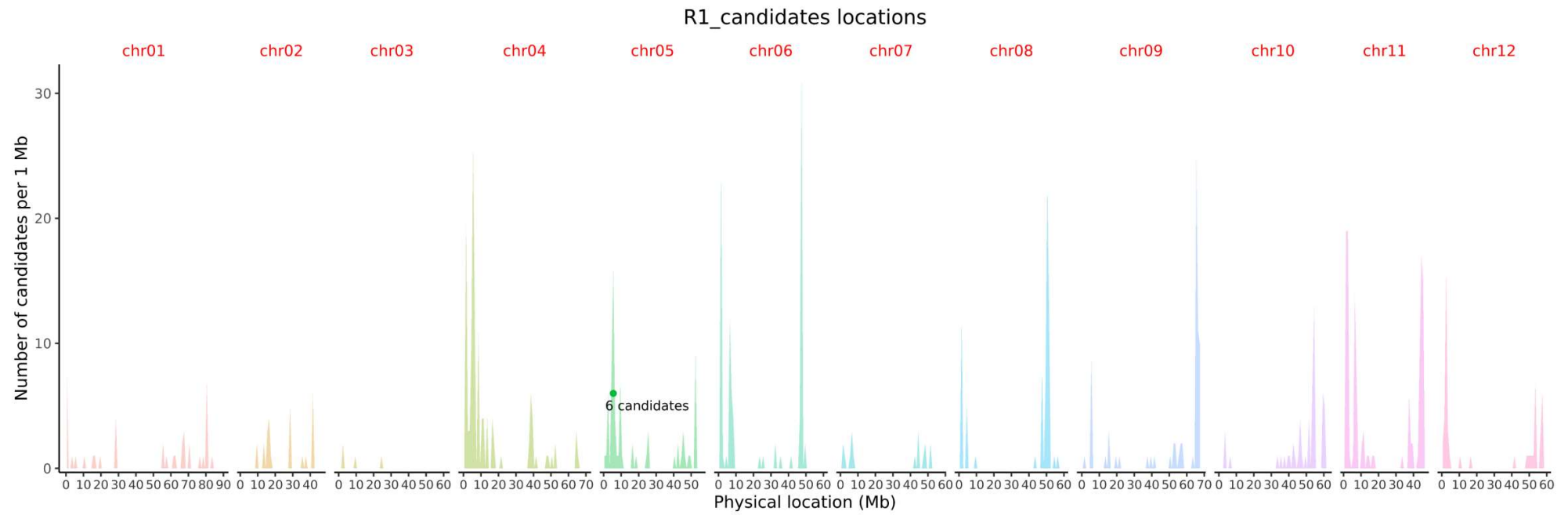

Supplement: Web_Material_uhad211 [file web_material_uhad211.zip › SF4_Supplementary Figure 4 R1.pdf]
